# A novel bispecific EGFR/Met antibody blocks tumor-promoting phenotypic effects induced by resistance to EGFR inhibition and has potent antitumor activity

**DOI:** 10.1038/onc.2013.245

**Published:** 2013-07-01

**Authors:** R Castoldi, V Ecker, L Wiehle, M Majety, R Busl-Schuller, M Asmussen, A Nopora, U Jucknischke, F Osl, S Kobold, W Scheuer, M Venturi, C Klein, G Niederfellner, C Sustmann

**Affiliations:** 1 Pharma Research and Early Development (pRED), Roche Diagnostics GmbH, Penzberg, Germany; 2 Division of Clinical Pharmacology, Department of Internal Medicine IV, Ludwig-Maximilians-Universität München, Munich, Germany; 3 pRED, Roche Glycart AG, Schlieren, Switzerland

**Keywords:** EGFR, Met, HGF, EGF, bispecific antibody

## Abstract

Simultaneous targeting of epidermal growth factor receptor (EGFR) and Met in cancer therapy is under pre-clinical and clinical evaluation. Here, we report the finding that treatment with EGFR inhibitors of various tumor cells, when stimulated with hepatocyte growth factor (HGF) and EGF, results in transient upregulation of phosphorylated AKT. Furthermore, EGFR inhibition in this setting stimulates a pro-invasive phenotype as assessed in Matrigel-based assays. Simultaneous treatment with AKT and EGFR inhibitors abrogates this invasive growth, hence functionally linking signaling and phenotype. This observation implies that during treatment of tumors a balanced ratio of EGFR and Met inhibition is required. To address this, we designed a bispecific antibody targeting EGFR and Met, which has the advantage of a fixed 2:1 stoichiometry. This bispecific antibody inhibits proliferation in tumor cell cultures and co-cultures with fibroblasts in an additive manner compared with treatment with both single agents. In addition, cell migration assays reveal a higher potency of the bispecific antibody in comparison with the antibodies' combination at low doses. We demonstrate that the bispecific antibody inhibits invasive growth, which is specifically observed with cetuximab. Finally, the bispecific antibody potently inhibits tumor growth in a non-small cell lung cancer xenograft model bearing a strong autocrine HGF-loop. Together, our findings strongly support a combination treatment of EGFR and Met inhibitors and further evaluation of resistance mechanisms to EGFR inhibition in the context of active Met signaling.

## Introduction

Escape mechanisms occurring in cancer cells and which develop in response to inhibition of a specific signaling pathway often limit efficacy of targeted single-agent therapies.^1^ Understanding the biology of such acquired but also intrinsic resistance mechanisms in tumors is pivotal for devising future rational combination therapies. The inhibition of a single receptor tyrosine kinase signaling presents a good example of molecular networks, which mediate tumor escape.^2^ A cross-talk of epidermal growth factor receptor (EGFR) and Met in transformed cells was already described in 2000 by Strom *et al.*^3^ EGFR is a member of the ErbB family of receptor tyrosine kinases consisting of EGFR (ErbB1), HER2/neu (ErbB2), HER3 (ErbB3) and HER4 (ErbB4).^4^ Constitutive EGFR signaling has a role in tumor biology by promoting survival and proliferation of cancer cells. Several EGFR-specific small molecular weight inhibitors (for example, gefitinib or erlotinib) as well as antibody modulators (cetuximab or panitumumab) have been developed and are approved for clinical use.^5^ Met and its ligand hepatocyte growth factor (HGF) are important mediators of tumor growth, survival and metastasis.^6, 7^ Similarly as for EGFR, a multitude of inhibitors, small molecules or monoclonal antibodies directed against Met (for example, tivantinib or onartuzumab (MetMAb)), are currently tested in clinical trials.^8^ Increased HGF/Met signaling can limit the effect of EGFR pathway inhibition and has been linked with acquired resistance to EGFR-targeted drugs in EGFR-mutant lung tumors.^9, 10^ Although the incidence of acquired resistance, as observed in non-small cell lung cancer, is only about 10%, Met is considered to be a major escape route for EGFR-targeted therapies.^11, 12^ Not surprisingly, ErbB family members may also confer resistance to Met tyrosine kinase inhibition.^13, 14^ Perturbation of both receptors' activity suggests that EGFR and Met signaling nodes are highly and dynamically interconnected.^15, 16^ These findings are further substantiated in various cellular models and as such, may reflect a general phenomenon.^17, 18, 19, 20, 21^ As murine HGF is only weakly cross-reactive to human Met, a combination of erlotinib and SGX523, a small molecule inhibitor of Met, was assessed in transgenic mice expressing human HGF and found to be superior to both single-agent treatment.^22^ In addition, results of a combination study of erlotinib and onartuzumab strengthen the co-targeting rationale.^23^ In this study, we demonstrate for the first time that, under conditions of active EGFR and Met signaling, treatment with specific EGFR inhibitors induces an increase in phosphorylated AKT and most importantly enhances the invasive properties of tumor cells. To test the hypothesis that combined inhibition of both receptor activities is required to suppress invasiveness, we generated a bispecific antibody based on the anti-EGFR antibody cetuximab and the Met-specific 5D5 antibody. The selected format for the bispecific antibody was that of a 2+1 molecule, which then allows fixed stoichiometry and consequent balanced inhibition of both receptors.

## Results

### EGFR inhibition triggers p-AKT and induces invasion in HGF-stimulated tumor cell lines

**Figure 1:**
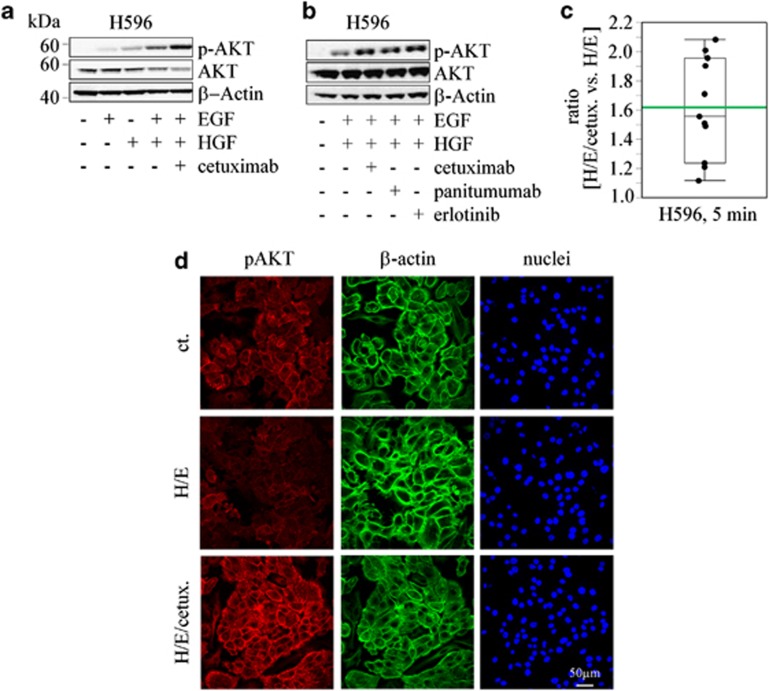
EGFR inhibition under EGF and HGF-stimulated conditions induces an increase in AKT phosphorylation. (**a**) AKT status in stimulated H596, in the presence or absence of cetuximab. (**b**) AKT status after treatment with cetuximab, panitumumab or erlotinib. (**c**) Box plot presentation of cetuximab-dependent pAKT stimulation. Analysis of the ratio of HGF/EGF (H/E) treatment versus H/E treatment in the presence of cetuximab (*n*=11 biological replicates). The box indicates 25th, 50th (median) and 75th percentiles, as well as mean (green bar). (**d**) Confocal microscopy at × 63 magnification of phospho-AKT and β-actin-stained A549 cells.

**Figure 2:**
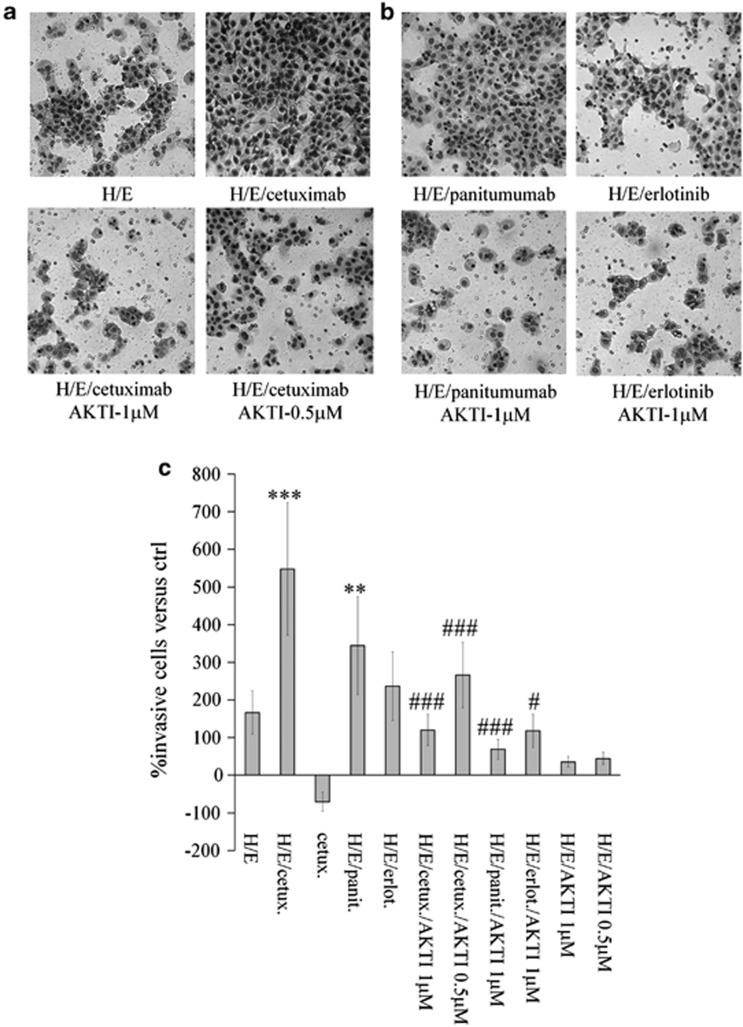
EGFR inhibition in the presence of HGF and EGF induces an invasive phenotype. (**a**, **b**) Invasive phenotype of HGF/EGF-stimulated A431 after treatment with cetuximab, panitumumab and erlotinib±AKTi -1/2VIII. (**c**) Quantitation of percentage invasive cells compared with untreated cells (statistics: treated versus untreated (*) or treated versus treated plus AKTi-1/2VIII (^#^) (^x^*P*<0.05; ^xx^*P*<0.01; ^xxx^*P*<0.001, where x, xx, xxx are either * or #)).

H596 cells, on stimulation with EGF and HGF and treatment with cetuximab, displayed an increase in phospho-AKT compared with untreated stimulated cells. This effect was also observed with the two alternative EGFR inhibitors panitumumab and erlotinib (Figures 1a and b). This observation, made consistently in the background of potent stimulation by HGF and EGF, was consistent and reproducible over a large set of experiments with a mean increase of 1.62 (Figure 1c). The EGFR inhibitors did not affect phosphorylation by themselves in the absence of EGF and HGF stimulation (Supplementary Figures S1A and B). Treatment with HGF and cetuximab led to a very modest increase of phospho-AKT in comparison with HGF-treatment only (Supplementary Figure S1C). Furthermore, spatially restricted increase of AKT phosphorylation was clearly observed in the membrane proximal region of A549-stimulated cells as described for H596 cells and in the context of EGFR inhibition (Figure 1d), which might be indicative for a potential role in migration and invasion events. In order to explore possible functional consequences, we tested the effect of EGFR inhibitors in an invasion assay using HGF and EGF-stimulated cells. Experiments were performed with A431 cells, as this cell line is a good model to study motility in Matrigel chambers, it responds to cetuximab treatment with an increase in phospho-AKT when stimulated with HGF and also displays increased invasion on treatment with HGF and/or EGF (data not shown). Cetuximab treatment after stimulation with EGF and HGF increased the invasive phenotype of A431 cells in a statistically significant manner (*P*<0.001) and this effect was dose-dependently reverted by co-treatment with an AKT inhibitor (AKTi-1/2 VIII; Figures 2a and c). A similar—albeit smaller—increase in invasion was induced by panitumumab and erlotinib treatments, and it was similarly impaired by the addition of an AKT inhibitor (Figures 2b and c). The AKT inhibitor was used at 1 μM: at this concentration it abrogated Ser473 phosphorylation, which is an activation marker, and was not cytotoxic in the assay (Supplementary Figures S2A and B).

### MetHer1 impairs proliferation in selected cell lines

To test the hypothesis whether the increase in phospho-AKT and the accompanying invasive phenotype, potentially mediating resistance to EGFR inhibition in the presence of HGF, could be reverted by the simultaneous inhibition of the HGF receptor Met, we generated a bispecific antibody construct capable of blocking EGFR and Met (MetHer1) (Supplementary Figures S3A–C). This was achieved by cloning the variable regions of cetuximab into an immunoglobulin G1 (IgG1) antibody backbone with a monovalent anti-Met single chain Fab similar to the one-armed 5D5 (onartuzumab) fused at the C-terminus of one of the heavy chains. Correct heavy chain hetero-dimerization was enforced using the knobs-into-holes technology.^24^ The final product had a purity >98% and was able to simultaneously bind to both antigens (Supplementary Figures S3D and E), displaying binding kinetics for each antigen in the nM range, comparable to those of the parental monospecific antibodies (Supplementary Figures S4A and B). As a side-product resulting in a bispecific antibody with two single chain Fab fusions would be agonistic, the activating marker phospho-Met was monitored in the presence of MetHer1 and in comparison with the bivalent and agonistic Met antibody. No agonism could be seen for MetHer1 (Supplementary Figures S3F).

**Figure 3:**
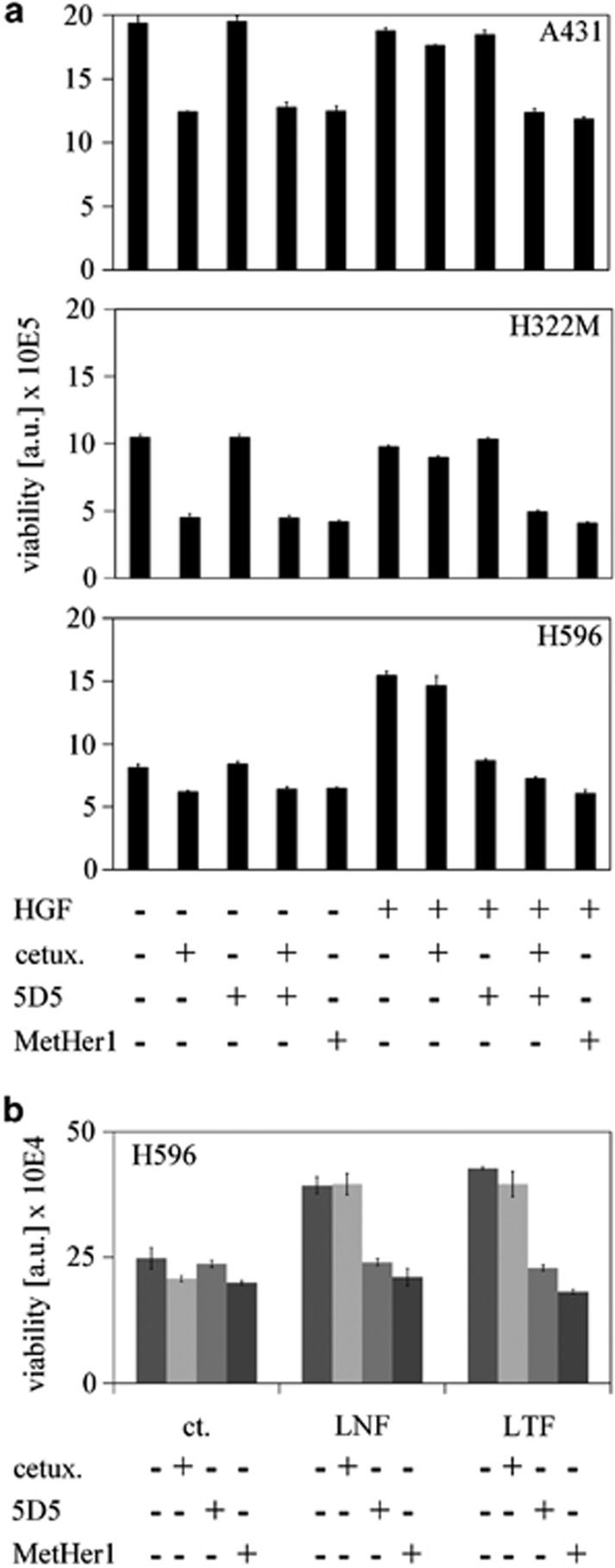
MetHer1 efficacy *in vitro*: effect on tumor cell proliferation. (**a**) Viability of indicated cell lines on antibody treatment. (**b**) Viability of H596 cultivated alone (ct.), or in the presence of normal (LNF) and tumor (LTF) lung fibroblasts. Cells were treated with MetHer1 and parental antibodies for comparison.

MetHer1 was further characterized *in vitro* for its effect on viability in basal conditions in A431, H596 and H322M cell lines and efficacy was compared with the two parental antibodies given as monotherapy or in combination (Figure 3a). Cells were cultivated in medium supplemented with 10% fetal calf serum (FCS) and HGF was added for comparison as it is essential for the functionality of the ligand-dependent 5D5 component of MetHer1. Treatment only with cetuximab was already efficacious in A431 cells, which are known to be EGFR addicted, but efficacy was completely lost on addition of HGF. In this setting, 5D5 antibody alone had no effect as well, whereas only MetHer1 or the combination of both parental antibodies induced a clear and significant reduction in cell viability (approximately 40%). This suggests that only inhibiting both receptors simultaneously may have therapeutic potential in tumor cells where both pathways are active. A very similar result was obtained with H322M, with MetHer1 showing a 60% growth inhibition. In this cell line as well, addition of HGF *per se* did not enhance proliferation, which 5D5 alone could also not block. However, addition of HGF impaired the anti-proliferative effect of cetuximab and only treatment with the combination of cetuximab and 5D5 or with MetHer1 restored growth inhibition. mRNA profiling data suggest a very low expression of Met in this particular cell line, compared with the other two (data not shown) and our results imply that the growth inhibition induced by MetHer1 occurred mainly via the EGFR-specific arm. Nevertheless, a comparable effect was not observed, when HGF-stimulated cells were treated with cetuximab alone.

In H596 cells stimulated with HGF, MetHer1 mediated 60% growth inhibition, which was significantly greater than that induced by 5D5 alone (*P*<0.001). Co-culture of H596 with normal and tumor lung fibroblasts resulted in a higher proliferation rate after 5 days, which was significantly reduced by treatment with 5D5 and MetHer1, but not by cetuximab (Figure 3b). The effect was probably dependent on fibroblasts producing HGF (Supplementary Figure S5A).

The anti-proliferative effect of MetHer1 was also evaluated in combination with a sub-optimal dose of the chemotherapeutic agent cisplatin in H596 and BxPC3. BxPC3 represents a pancreatic model in which the bispecific showed only a weak effect on viability (Supplementary Figure S5B). Nevertheless, combined treatment was superior to the effect of cisplatin alone (*P*<0.001) with an overall percentage growth inhibition of >60%. A combination of MetHer1 and cisplatin in H596, which already responded well to MetHer1 mono-treatment, had no additional effect. This supports the rationale that a combination of bispecific antibody with reduced and thus better tolerated doses of a chemotherapeutic can improve efficacy and safety, particularly in tumor models, which are less dependent on signaling (that is, BxPC3).

### MetHer1 prevents HGF-induced scattering

**Figure 4:**
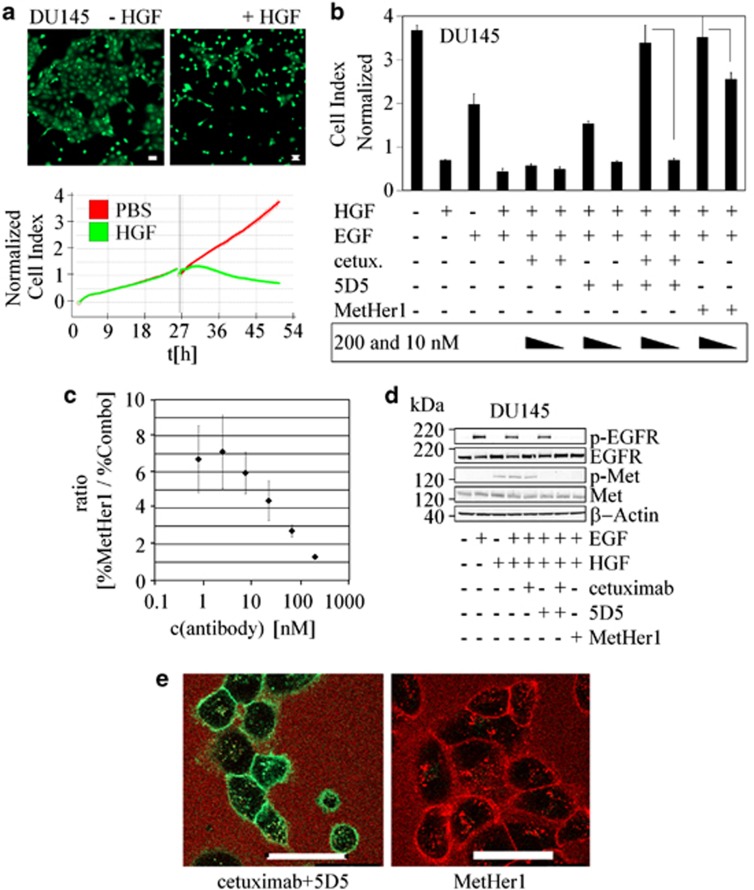
MetHer1 effect on HGF-induced motility. (**a**) DU145 after 24-h treatment with 30 ng/ml HGF. Confocal microscopy analysis of calcein-stained cells and effect on impedance measured by RTCA (white bar x, y: 50 μm). (**b**) Quantitation of MetHer1 effect on HGF-induced DU145 scattering. (**c**) Dose-response curve analysis of scatter assay in DU145. The efficacy of bispecific antibody and cetuximab+5D5-mediated inhibition of cell dissemination was determined after 24 h and the ratio of both calculated. (**d**) Basal and on-treatment receptor status of EGFR and Met. (**e**) Internalization of fluorescently labeled antibodies evaluated in DU145 cells after 4 h of incubation (white bar x, y: 50 μm).

HGF is also a known motility factor, which induces scattering and invasion of epithelial cells. This is phenotypically characterized by a change in cell shape and the effect can be macroscopically observed in Figure 4a showing DU145 after 24 h of treatment with HGF. Cellular migration can be semi-quantitatively evaluated with a real-time cell analyzer (RTCA system), which measures impedance changes as surrogate parameter of cell adhesion. As reported in Figure 4a, HGF-induced cell motility and dissemination of DU145 cells, thus reducing the measured impedance, when compared with control. Scattering was quantified in a graph where a normalized cell index (compound addition) was plotted against time. DU145 were treated with cetuximab and 5D5, the combination of both and MetHer1 (at 200 and 10 nM) and stimulated with EGF and HGF. At high dose, MetHer1 could completely revert the HGF-induced scattering and to a smaller extent also at the low dose. In the latter case, no efficacy was seen instead for the combination of the monospecific antibodies. Efficacy of 5D5 alone was reduced by the influence of EGF treatment, which *per se* also showed an effect on cell adhesion (Figure 4b). Viability analysis displayed no differences between treatments, excluding any influence of cell viability or proliferation on the interpretation of the results (data not shown). A human IgG control antibody did not influence cellular scattering (Supplementary Figures S6C and D), suggesting specificity of the reported data. The potential superiority of MetHer1 at low doses was further evaluated in a dose-response scatter experiment. The percentage scatter inhibition for MetHer1 or the combination (Combo) was calculated and the ratio of both determined. MetHer1 displayed superior inhibitory activity over three logs of antibody concentration with a sevenfold higher potency at doses as low as 1 nM (Figure 4c).

To better assess the superiority of MetHer1 versus the combination in preventing growth factor-induced cell dissociation at a low dose, the kinetics of internalization of the two single agents in comparison with MetHer1 was evaluated in a fluorescence-activated cell sorting assay. Presence of the receptors on the cell surface was measured after binding with the respective antibodies for 2 h, versus t0 (Supplementary Figure S6A). The amount of antigen–antibody complex on the cell surface was unchanged within this time. Intracellular staining was only visible as speckle-like structures after 4 h of incubation with fluorescently labeled antibodies by confocal microscopy (Figure 4e, Supplementary Figure S6B). Cetuximab binding appeared to be stronger compared with 5D5, which may be a consequence of differential antigen expression (Figure 4d). There was no difference in the kinetics of internalization between the molecules. Therefore, superiority of MetHer1 in the scatter assay could not be explained by differential internalization.

### MetHer1 inhibits EGFR and Met-related pathways

**Figure 5:**
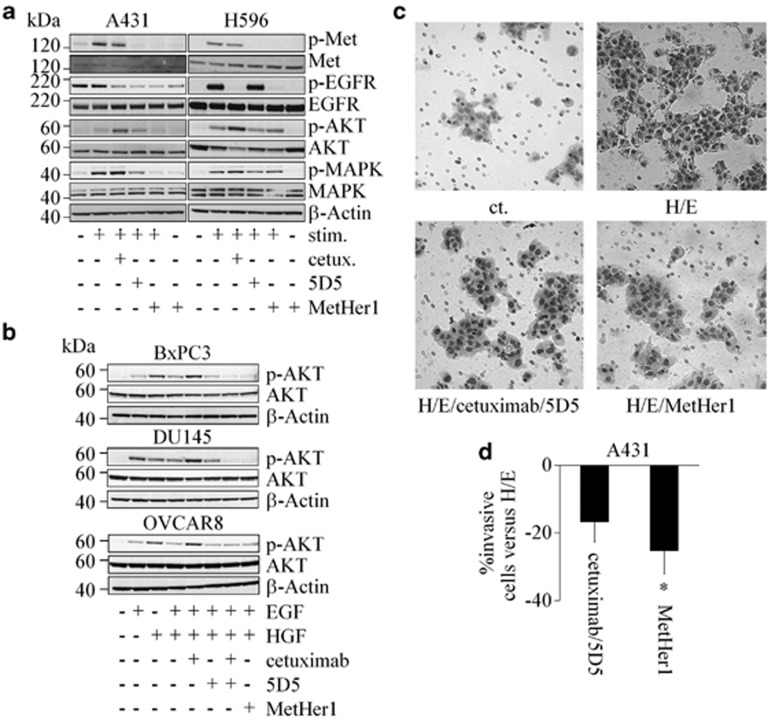
MetHer1 inhibits downstream signaling and invasion. (**a**) Expression and phosphorylation status of indicated proteins in A431 and H596 on treatment. A431 were stimulated with HGF, H596 with HGF and EGF. (**b**) Phosphorylation status of AKT in indicated cell lines after antibody treatment. (**c**) Invasive A431 cells after treatment with MetHer1 (H/E=HGF and EGF). (**d**) Percentage of invasive A431 after stimulation with HGF/EGF and treatment with indicated antibodies. *P*-values were calculated versus stimulated cells; *P<0.05.

MetHer1 efficacy in proliferation experiments was accompanied by a strong decrease of target phosphorylation in A431 and H596 (Figure 5a), as well as in other *in vitro* models (Supplementary Figure S7A). In A431, phospho-ERK1/2 was blocked by MetHer1 but not or only minimally by treating with the single parental antibodies. The level of phospho-AKT, which was found to be increased in HGF/EGF-stimulated cells after treatment with cetuximab alone reverted back to basal untreated values in the presence of MetHer1 in five cancer cell lines of different tissue origins (Figures 5a and b). In BxPC3, we observed phosphorylation of Met after stimulation of cells with EGF, which might be due to a cross-talk between EGFR and Met. MetHer1 also reduced invasion induced by HGF and EGF and significantly counteracted the effect induced by cetuximab parental antibody in equal settings (Figures 5c and d). The effect of simultaneous treatment with cetuximab and 5D5 is additionally shown for comparison.

### MetHer1 has a potent antitumor effect *in vivo*

**Figure 6:**
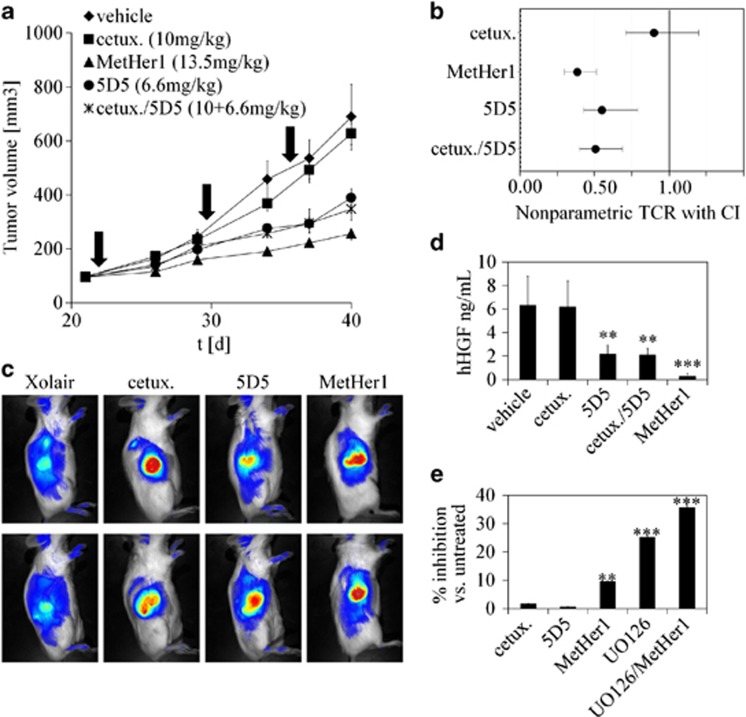
MetHer1 is efficacious *in vivo* in a HGF-overexpressing A549 human lung adenocarcinoma xenograft model. (**a**) Mean tumor volume (arrows=treatment). The anti-IgE antibody Xolair was used as control antibody. (**b**) Nonparametric treatment-to-control-ratio (TCR) of tumor growth inhibition at the end of study. (**c**) *In vivo* imaging in two representative animals per group. (**d**) Quantitation of human HGF from serum samples at the end of study. (**e**) Growth inhibition of A549 cl.20 *in vitro*, with a sub-optimal dose of the MEK inhibitor UO126 and indicated antibodies (**P<0.01, ***P<0.001).

To test the efficacy of MetHer1 in a mouse model, an A549 tumor cell line overexpressing HGF was generated by viral transduction with a vector-encoding human HGF to overcome the issue of non-cross-reactivity of murine HGF to human Met and ensure an efficacy contribution by the 5D5 component. Several clones were generated and their ability to produce HGF in the presence and absence of selection pressure was evaluated by enzyme-linked immunosorbent assay over a period of 29 days to ascertain stable expression (Supplementary Figure S8A). Clone20 was selected because of high secretion levels of HGF and constitutive Met phosphorylation (Supplementary Figures S8A and C). The RTK signaling network in this clone was compared with parental A549 by using a phospho-RTK array and affymetrix profiling. Overall, A549 clone20 was comparable in its mRNA expression profile but displayed a slightly different activation pattern of receptor tyrosine kinases (Supplementary Figure S8B and data not shown). HGF-producing A549 clone20 was characterized by cell surface binding of fluorescently labeled MetHer1, 5D5 and cetuximab. Although cetuximab and MetHer1 displayed a strong binding capacity, 5D5 binding was found to be reduced in the HGF-overexpressing clone compared with un-transduced cells (Supplementary Figure S8E). This might be a consequence of competition with ligand and/or lower steady-state Met cell surface expression levels because of constitutive internalization induced by the ligand HGF (Supplementary Figure S8C). MetHer1 inhibited *in vitro* phosphorylation of both EGFR and Met to the same extent as the parental antibodies. When subcutaneously implanted into mice, tumors produced HGF (7.4±2.71 ng/ml: average of 10 animals), which was further confirmed *ex vivo*, in tumor lysates (Supplementary Figure S8D). MetHer1 efficacy was tested *in vivo* in the subcutaneous setting and compared with the parental antibodies, which were administered in an equimolar ratio as monotherapy or in combination. Tumor growth inhibition at the end of study was with 75% higher for MetHer1 but not statistically significantly different from the combination (55%) after three weekly cycles of treatment (tumor growth inhibition for cetuximab and 5D5: 11% and 51%). Data are presented as tumor growth inhibition and nonparametric treatment-to-control-ratio graph (Figures 6a and b). Near infrared fluorescence analysis with fluorescently labeled antibodies confirmed *in vivo* binding, as shown with two representative animals per group (Figure 6c). Human HGF measured in the tumors was strongly reduced in the MetHer1 treatment group compared with the vehicle group, probably as a consequence of smaller tumor sizes (Figure 6d). The low efficacy observed after treatment with cetuximab was expected because of mutant KRAS status. To predict the effect of a putative combination of MetHer1 with a MEK inhibitor, which would block the pathway downstream of KRAS, the effect of MetHer1 and the MEK inhibitor UO126 on proliferation was tested *in vitro* in A549 clone20 cells. Figure 6e shows the results obtained when UO126 was administered at the sub-optimal dose of 5 μM alone or in combination with MetHer1 (UO126 IC50 for this cell line: 12.7 μM; data not shown). In combination with the MEK inhibitor, a fourfold increase in the percentage inhibition was observed, supporting that the KRAS mutation strongly influences treatment efficacy.

## Discussion

In this study, we investigated in detail the counterbalancing mechanisms mediated by Met that confer resistance to targeted inhibition of EGFR. We confirmed in tumor cell lines from different origins that treatment with EGFR inhibitors results in a transient upregulation of phospho-AKT under conditions of co-activation of the EGFR and Met pathways. In the presence of active Met signaling, EGFR inhibition also enhanced invasiveness (Figure 2a). Invasive growth of tumor cells on stimulation with EGF or HGF is well known.^25^ Although a variety of studies on the cross-talk of the two receptors and their inhibition have been published,^19, 21, 26^ it has not been previously described that addition of EGFR inhibitors to HGF-stimulated cells can increase invasiveness in comparison with growth factor treatment only. Bonine-Summers *et al.*^27^ previously published that the EGFR inhibitor gefitinib also inhibits Met signaling, which is in contrast to our findings. It has been shown that gefitinib very potently targets cyclin-G-associated kinase also.^28^ Meanwhile, it is known that cyclin-G-associated kinase regulates PP2A and clathrin-mediated endocytosis, both also important for Met regulation, which might explain the authors' findings.^29, 30^ A very comprehensive study by Gusenbauer *et al.*^31^ demonstrates the intricate cell surface network for EGFR and Met but also for a variety of membrane proteins, which are involved in this signaling node. Interference by our EGFR inhibitors, especially antibodies binding EGFR, might shift the balance between these signaling nodes and thus produce the observed effects.

Maseki *et al.*^32^ reported that gefitinib-resistant head and neck squamous cell carcinoma can acquire an epithelial to mesenchymal transition phenotype, which is accompanied by an increase of phospho-AKT. A similar epithelial to mesenchymal transition process might occur in our experimental setting, accompanied by Twist and Snail-mediated repression of E-cadherin.^33^ Alternatively, phosphatidylinositol 3 kinase/AKT signaling could directly act on focal adhesion kinase.^34^ Focal adhesion kinase and Src are known to modulate E-cadherin and thereby promote cancer cell invasion.^35^ Further addition of an AKT inhibitor reversed the invasive phenotype similarly to the combined inhibition of EGFR and Met (Figures 2a, 5c and 5d). This implies, but does not unambiguously prove, that the transient increase of phospho-AKT is causally linked to the increase in invasiveness. In this context, it is an intriguing recent experimental finding that an artificial increase of phospho-AKT results in loss of cetuximab sensitivity in various lung cancer cell lines.^36^

Our findings could be clinically relevant in the setting of an adjuvant anti-EGFR therapy given that, independently from the well-known autocrine or paracrine HGF supply by tumor cells and/or tumor associated fibroblasts, it has been shown that HGF serum levels are elevated after surgery as part of the wound-healing process.^37, 38, 39^ However, the duration of this process in patients is unclear. Targeted EGFR inhibition in lung cancer, in an adjuvant setting, has already been studied.^40^ In 2002, the JBR.19 trial investigated gefitinib as maintenance treatment in resected non-small lung cancer. However, this trial was prematurely stopped because of negative results of the ISEL and SWOG 0023 trials with gefitinib. The ongoing RADIANT trial with erlotinib is primed to demonstrate whether EGFR inhibition in the adjuvant setting is beneficial.

Clinical trials with combinations of EGFR and Met inhibitors are ongoing. In this co-targeting setting, our data suggest that an imbalance of EGFR and Met-targeting activities in tumor samples may pose the risk of increased tumor spread. This could be of special concern if low molecular weight and antibody inhibitors with different pharmacodynamics are co-administered, thus making a stronger case for the development of the bispecific antibody we described. We have generated a bispecific antibody consisting of cetuximab and 5D5 in a 2+1 format under the assumption that a fixed stoichiometry of both targeting compounds should ensure simultaneous inhibition of both targets even in poorly accessible solid tumors. Mechanistically, such a bispecific antibody might for instance display differential avidity, clustering and internalization or antibody-dependent cell-mediated cytotoxicity properties in comparison with the combination of two antibodies. To our knowledge, MetHer1 presents the first bispecific IgG-like antibody targeting Met and EGFR. The antibody is non-agonistic and proves the concept of targeting both receptors simultaneously with a bispecific antibody. Previously, bispecific antibodies targeting EGFR and IGF-1R have been described with a similar co-targeting approach^41, 42, 43, 44^ as well as EGFR was used as targeting moiety for effector cell recruitment or payload delivery.^45, 46^ MetHer1 displays no agonistic activity in cellular assays and the overall activity was mostly similar to the combination of the parental antibodies cetuximab and 5D5. We observed differences in cell dissemination in the presence of low inhibitor concentrations. This could possibly be explained by an avidity effect, which raises the local Met inhibitor concentration and thereby enhances efficacy. We propose that in the presence of EGFR binding, the Met component of MetHer1 is enriched on the cell membrane and can better inhibit Met activity. A close proximity of both receptors has been previously shown by co-immunoprecipitation.^3^

In a ligand-dependent animal model, the overall activity of MetHer1 was superior but not significantly better than the combination of the parental antibodies cetuximab and 5D5 (Figure 6a). Efficacy of cetuximab is greatly impaired by the KRAS mutation found in A549. Although the mAb does not confer much antitumor activity, in the MetHer1construct, cetuximab could function as targeting moiety leading to more efficient 5D5 recruitment. This could explain the modest superiority of MetHer1 over the combination of parental antibodies but needs further investigation. The hypothesis is supported by our *in vitro* cell dissemination experiments whereby at lower doses MetHer1 was also more efficacious than the parental antibody combination (Figures 4b and c).

In contrast to onartuzumab, MetHer1 is a fully glycosylated human IgG1 antibody. Thus, MetHer1 retains effector function abilities and these are not affected by the C-terminal fusion of the 5D5 single chain Fab (data not shown). Cetuximab and 5D5, as used herein, also have a glycosylated human IgG1 Fc-part. In the A549 clone20 *in vivo* model, immune effector functions, for example, by residual macrophages, may have a role. However, theoretically, these effects should be stronger in the combination group, as the total Fc load per tumor cell is presumably higher than for the MetHer1 group.

Although inhibition of tumor growth is a primary parameter, it remains to be shown if the number of metastases is affected in models, which display stronger tumor spread, especially after excision of the primary tumor. A major hurdle is the availability of human HGF to activate Met in such a model. Transgenic mice producing human HGF have been described in the past and might help to address this problem.

In summary, the findings reported here highlight the complexity of perturbing regulatory networks by the use of targeted therapies, especially if multiple activating signals are present, which is the case in the majority of solid tumors, either *de novo* or as consequence of acquired resistance. Bispecific antibodies—as exemplified by MetHer1—facilitate targeting of two pathways without the risk of under-dosing one compound, efficiently counteract resistance mechanisms at the molecular level and yet retain the ability to effectively mediate antibody effector functions. Potential liabilities of such bispecific antibodies, for instance, cumulative toxicities or unanticipated modes of action, would need to be carefully evaluated during the development process.

## Materials and methods

### Cell culture

A431, A549 and BxPC3 were obtained from ATCC (Manassas, VA, USA); DU145, OVCAR8 and H322M from the NCI (Bethesda, MD, USA); H596 from Chugai Pharmaceuticals Co., Ltd. (Tokyo, Japan) and lung normal and tumor fibroblasts from Asterand plc (Royston, Herts, UK). Except H596, all cells were maintained in RPMI-1640 medium, supplemented with 10% FCS, non-essential amino acids, sodium pyruvate and L-glutamine (Gibco, Darmstadt, Germany). H596 were maintained in RPMI high glucose, supplemented with L-glutamine, 1 mM sodium pyruvate, 10 mM HEPES (PAN Biotech, Aidenbach, Germany) and 10% FCS. Cells were propagated according to standard cell culture protocols.

### Proteins and inhibitors

The variable heavy and light chain domain sequences of cetuximab and 5D5.v2, herein referred as 5D5, were cloned based on published sequences via gene synthesis in mammalian expression vectors. For cetuximab, a human IgG1 framework and kappa light chain backbone was used. For 5D5, two heavy chain 5D5 plasmids were used which carried the knobs-into-hole mutations^47^ and in which one was missing the VH-CH1 domain. MetHer1 was constructed from cetuximab with a human IgG1 backbone with knobs-into-hole and a single chain Fab fusion of Met at the knob heavy chain. Light and heavy chains were co-transfected in HEK-293F (Invitrogen/Life Technologies GmbH, Darmstadt, Germany) resulting in full glycosylation of all antibodies, then purified as previously described.^45^ Purity was analyzed using an Agilent HPLC 1100 (Agilent Technologies, Oberhaching, Germany) with a TSK-GEL G3000SW column (Tosoh Corp., Tokyo, Japan). Identity was confirmed by mass spectrometry and binding properties characterized by surface plasmon resonance (SPR). Cetuximab parental antibody was purchased from Merck Serono (Darmstadt, Germany), panitumumab from Amgen Inc. (Thousand Oaks, CA, USA). Met and EGFR ectodomains were transiently expressed and purified from HEK-293F supernatants. Recombinant huHGF and huEGF were obtained from R&D Systems (Minneapolis, MN, USA) and Gibco. AKTi-1/2 VIII and UO126 were bought from Calbiochem/Merck KgaA (Darmstadt, Germany). Other antibodies: pEGFR, pAKT1 (Epitomics, Burlingame, CA, USA), EGFR (Millipore/Merck KgaA, Darmstadt, Germany), pMet, Met, pMAPK, MAPK, AKT (Cell Signaling Technology Inc., Danvers, MA, USA) and β-actin (Abcam, Cambridge, UK).

### Immunoblot

Cells (5–8 × 10^5^ per well) were seeded in a six-well plate in medium with 0.5% FCS and treated the following day with 0.07 μM of cetuximab, panitumumab, 5D5 and MetHer1 and 5 μM erlotinib for 30 min (1 h for erlotinib) prior stimulation (HGF 30 ng/ml and EGF 50 ng/ml). After 5 or 15 min of incubation at 37 °C, cells were washed with phosphate-buffered saline, lysed and subjected to immunoblot analysis. For statistical analysis, a box plot analysis was applied.

### Invasion assay

A431 (50 000 cells per well) were pre-incubated for 15 min at 37 °C with 0.2 μM antibodies, 5 μM erlotinib or AKT inhibitor in medium with 0.5% FCS and seeded in Matrigel chambers (BD Biocoat Matrigel Invasion Chambers, BD Biosciences, Heidelberg, Germany), which were beforehand rehydrated and immersed in 24-well companion plates in medium with 10% FCS and/or growth factors plus treatment. HGF and EGF were added in the chambers before incubation for 43 h at 37 °C. Non-invading cells were removed from the upper surface of the membrane by scrubbing and cells were fixed and stained (Diff-Quick stain). Pictures were taken at a magnification of × 100 and invasive cells counted in four different fields each of quadruplicate membranes of two independent experiments. Standard deviation was calculated as average of all values. In parallel, 100 μl of the medium were used for a cytotoxicity assay (Promega, Madison, WI, USA) according to the manufacturer's instructions.

### Proliferation assays

Cells (A431, H322M: 2500 cells per well; H596: 5000 cells per well; A549 clone20: 1000 cells per well) were seeded in medium with 10% FCS and treated the following day with 0.2 μM of the antibodies for 15 min before stimulation with HGF 30 ng/ml. Viability was measured via Cell Titer Glo (Promega) at 5 days (A431, H322M and H596) and 4 days after treatment for A549 clone20. UO126 was added at 5 μM 24 h before measuring.

### Migration assay

Changes in cell morphology were monitored using xCelligence (Roche Applied Science, Mannheim, Germany). DU145 (3000 cells per well) were seeded in a 96 well-E-plate in medium supplemented with 0.5% serum and treated the following day with antibodies (200 and 10 nM) for 15 min before HGF and EGF stimulation (30 and 50 ng/ml).

### Xenograft study

To generate primary tumors, 1 × 10^7^ tumor cells in a volume of 100 μl phosphate-buffered saline were injected subcutaneously into the right flank of the mice. Animals were controlled 5 × per week for their health status. Tumor dimensions were measured by caliper on the staging day, and twice weekly for the treatment period. Animals were treated on study day 21, 28 and 35. All experiments were approved by the local regulatory agency. Nonparametric treatment-to-control-ratios based on end point analysis and the two-sided nonparametric confidence intervals compared with vehicle group were calculated to assess statistical significance.
