# Supplementary figures and images for: A novel bispecific EGFR/Met antibody blocks tumor-promoting phenotypic effects induced by resistance to EGFR inhibition and has potent antitumor activity
Source: Oncogene. 2013 Jul 1;32(50):5593–601. doi: 10.1038/onc.2013.245 (PMC3898114; doi:10.1038/onc.2013.245)

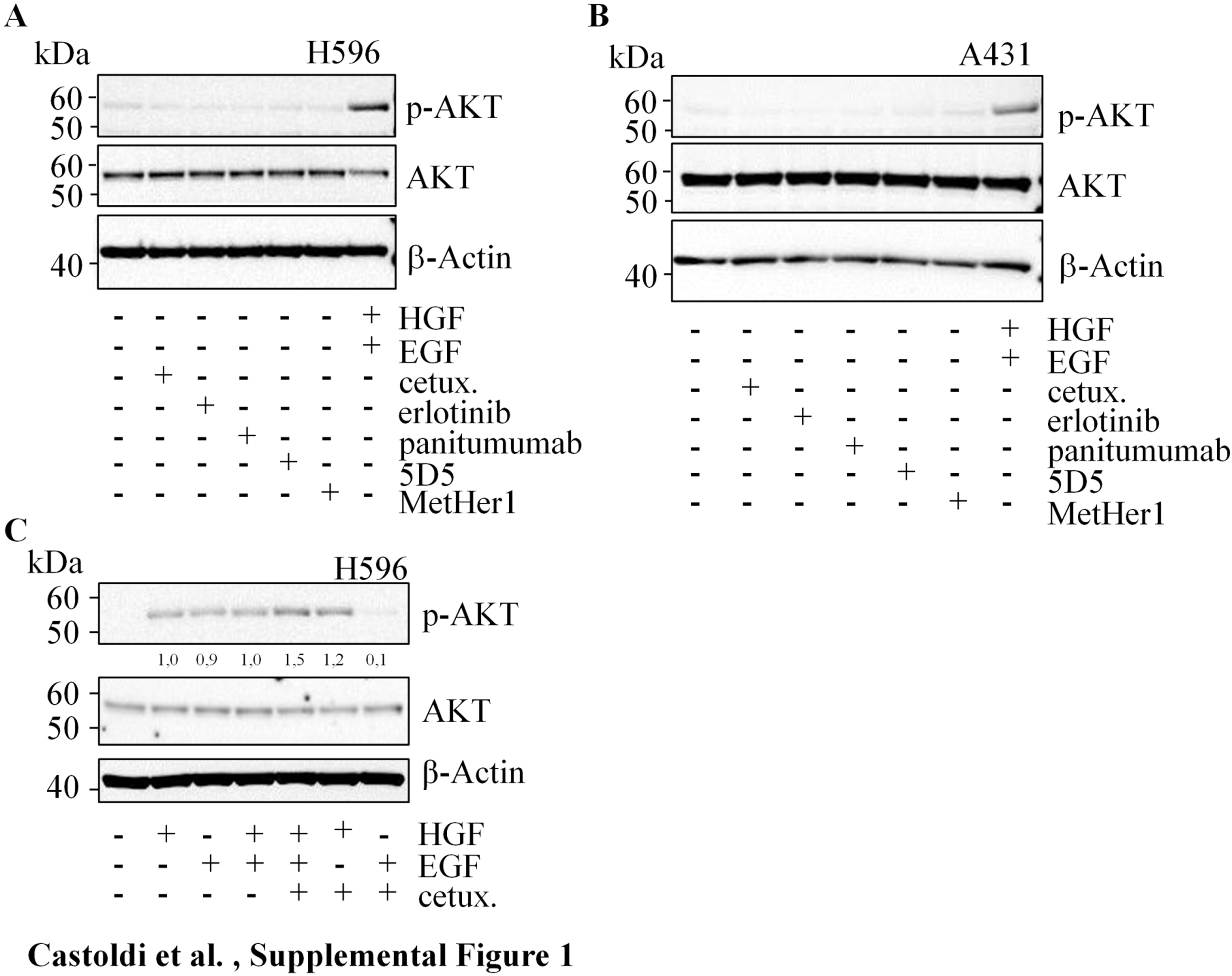

Supplement: Supplementary Figure S1 [file onc2013245x1.tif]

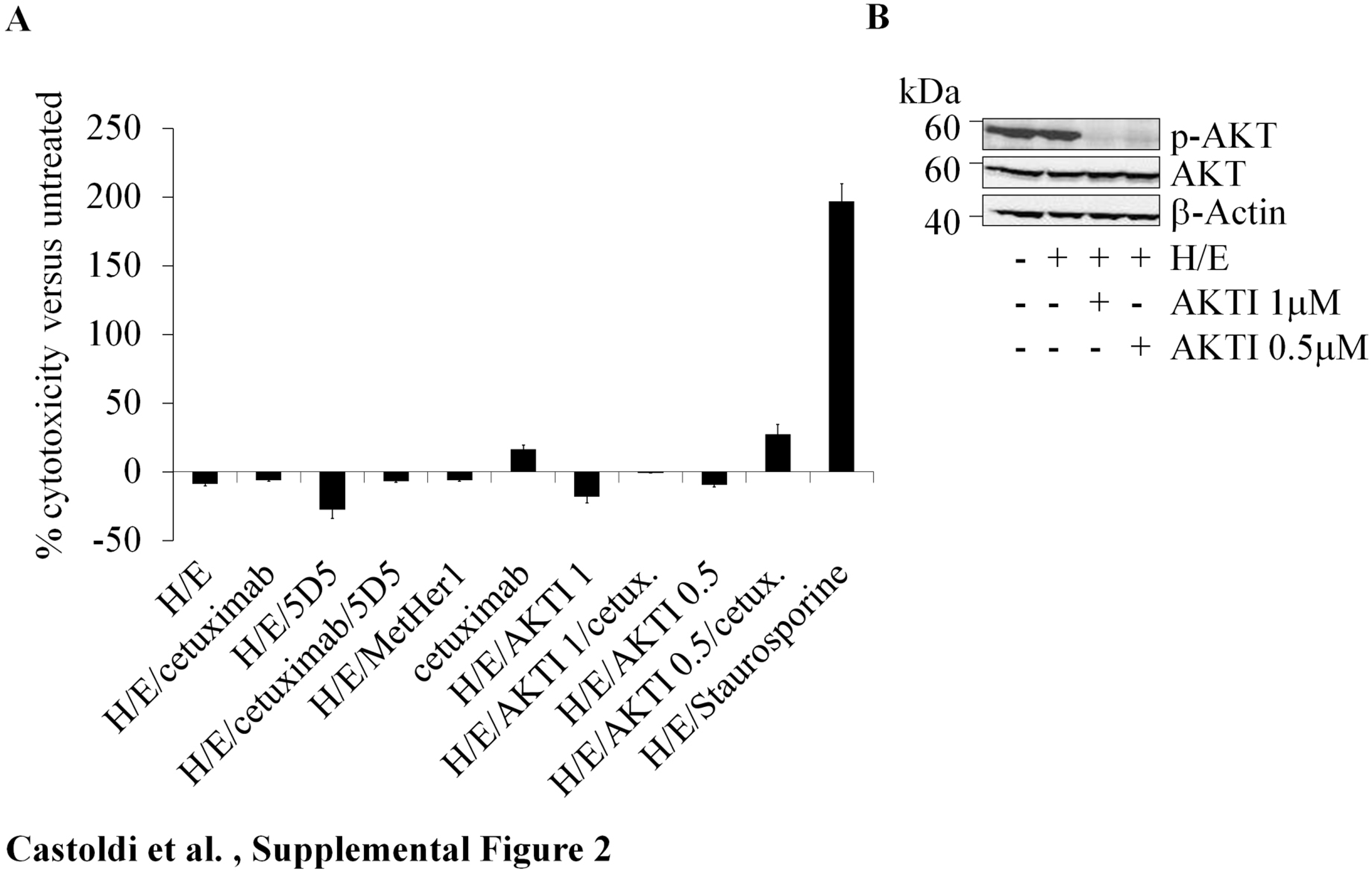

Supplement: Supplementary Figure S2 [file onc2013245x2.tif]

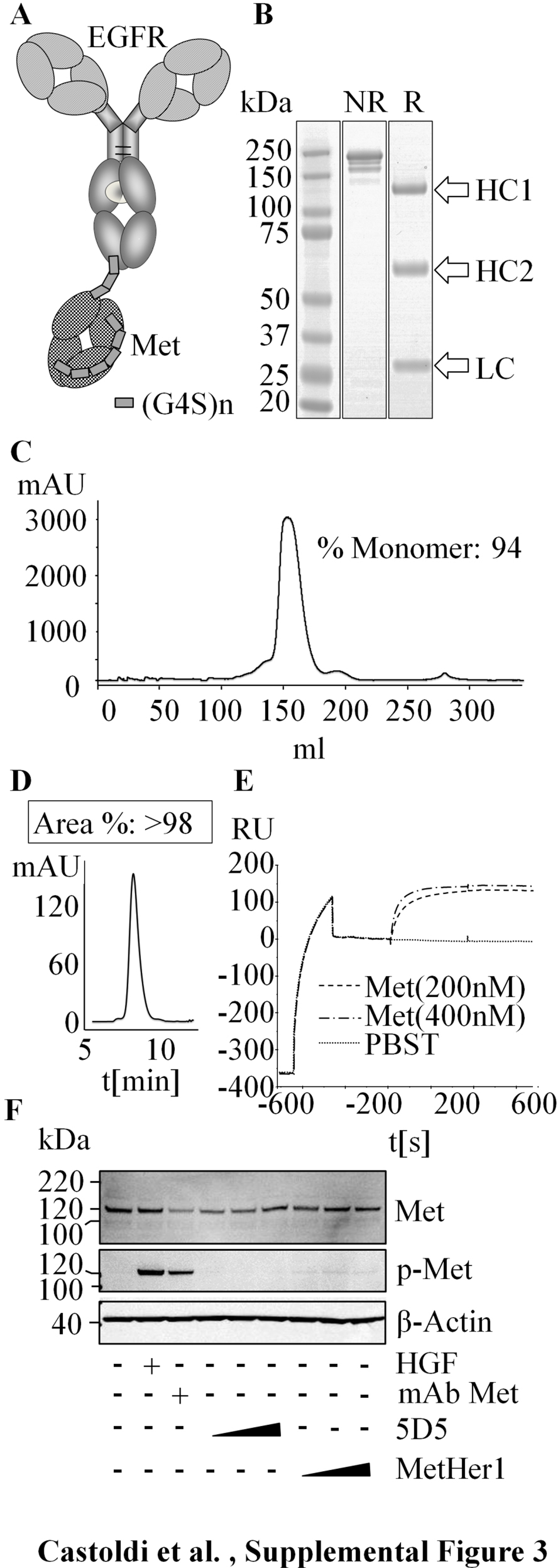

Supplement: Supplementary Figure S3 [file onc2013245x3.tif]

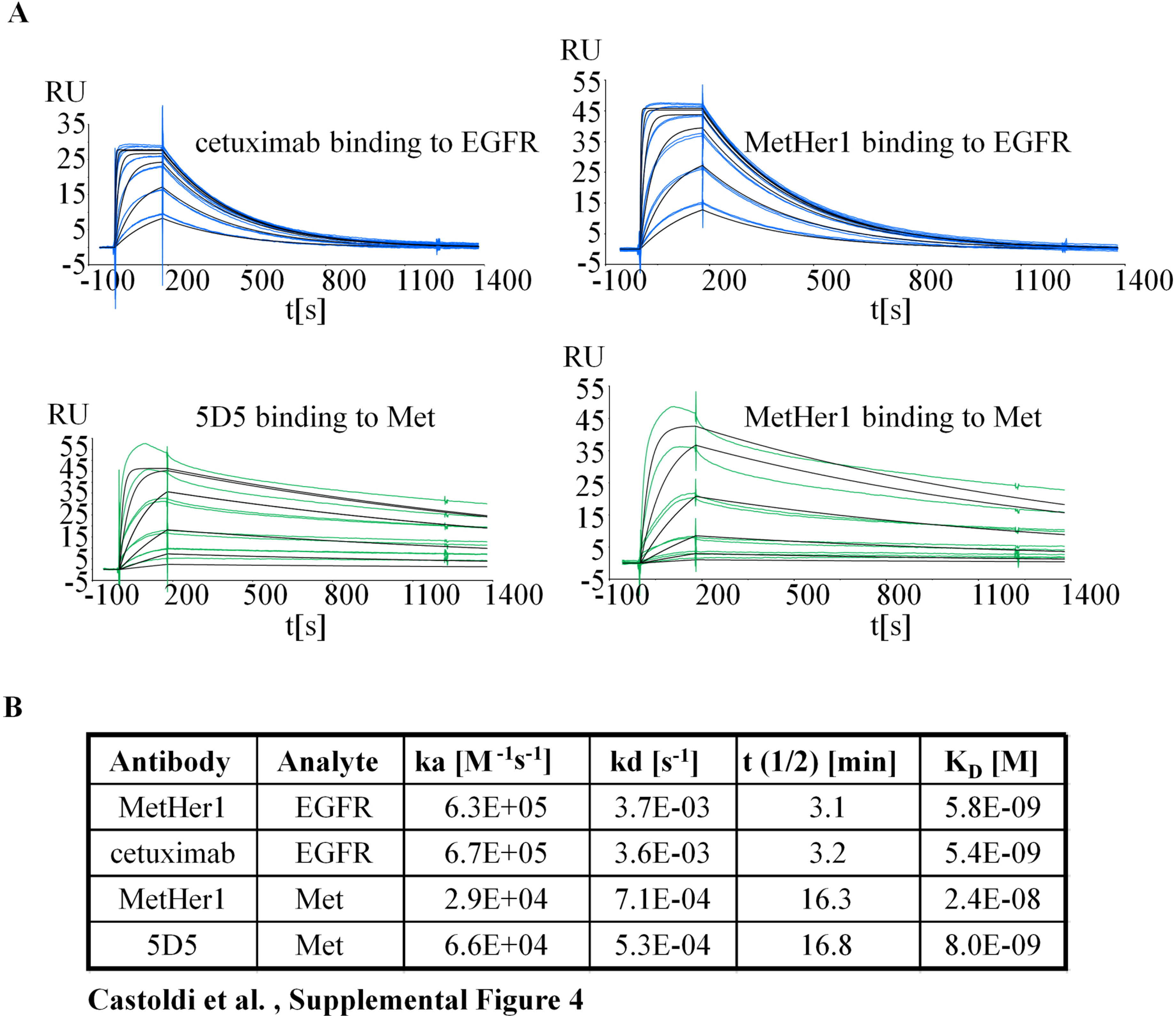

Supplement: Supplementary Figure S4 [file onc2013245x4.tif]

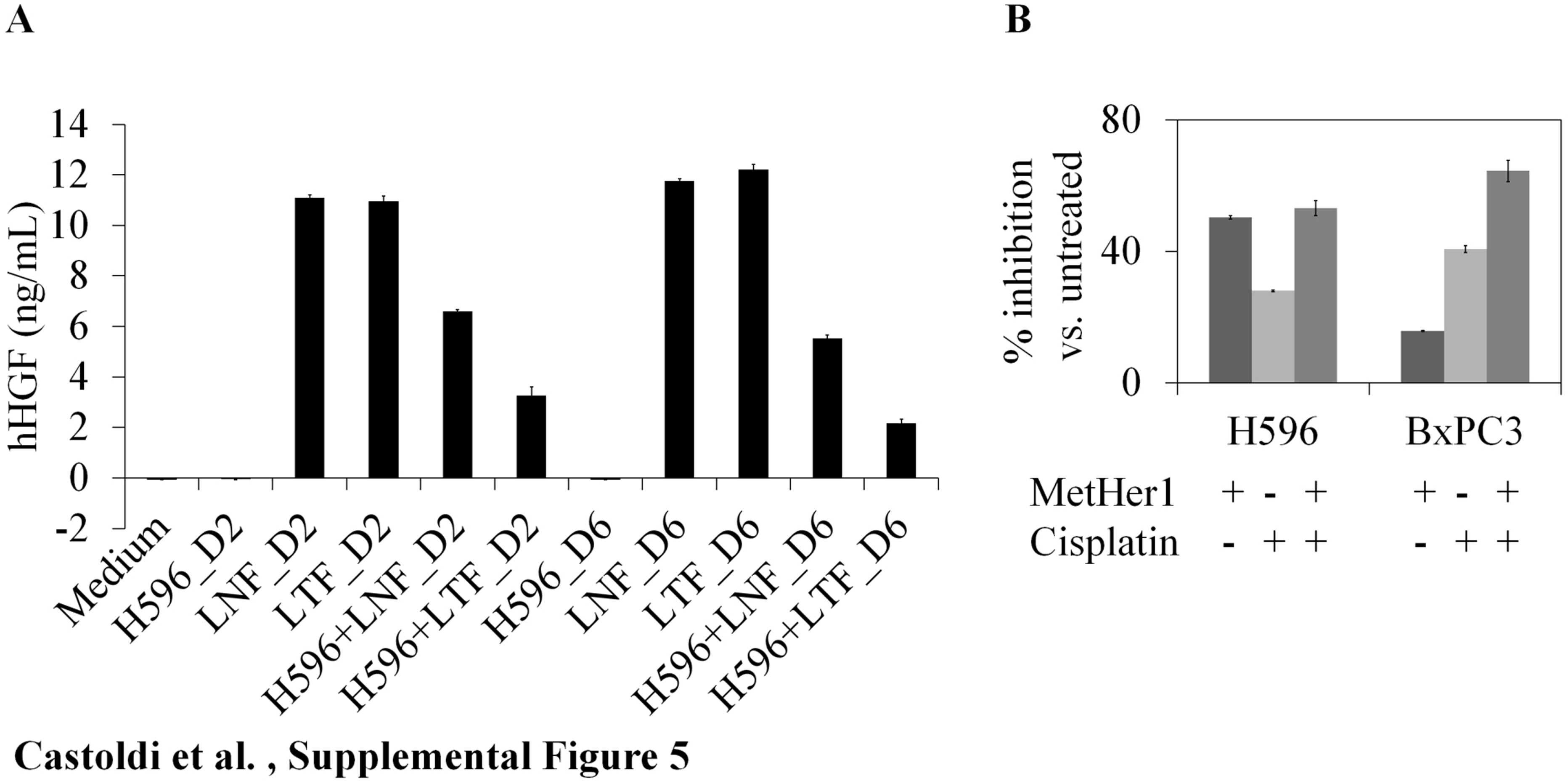

Supplement: Supplementary Figure S5 [file onc2013245x5.tif]

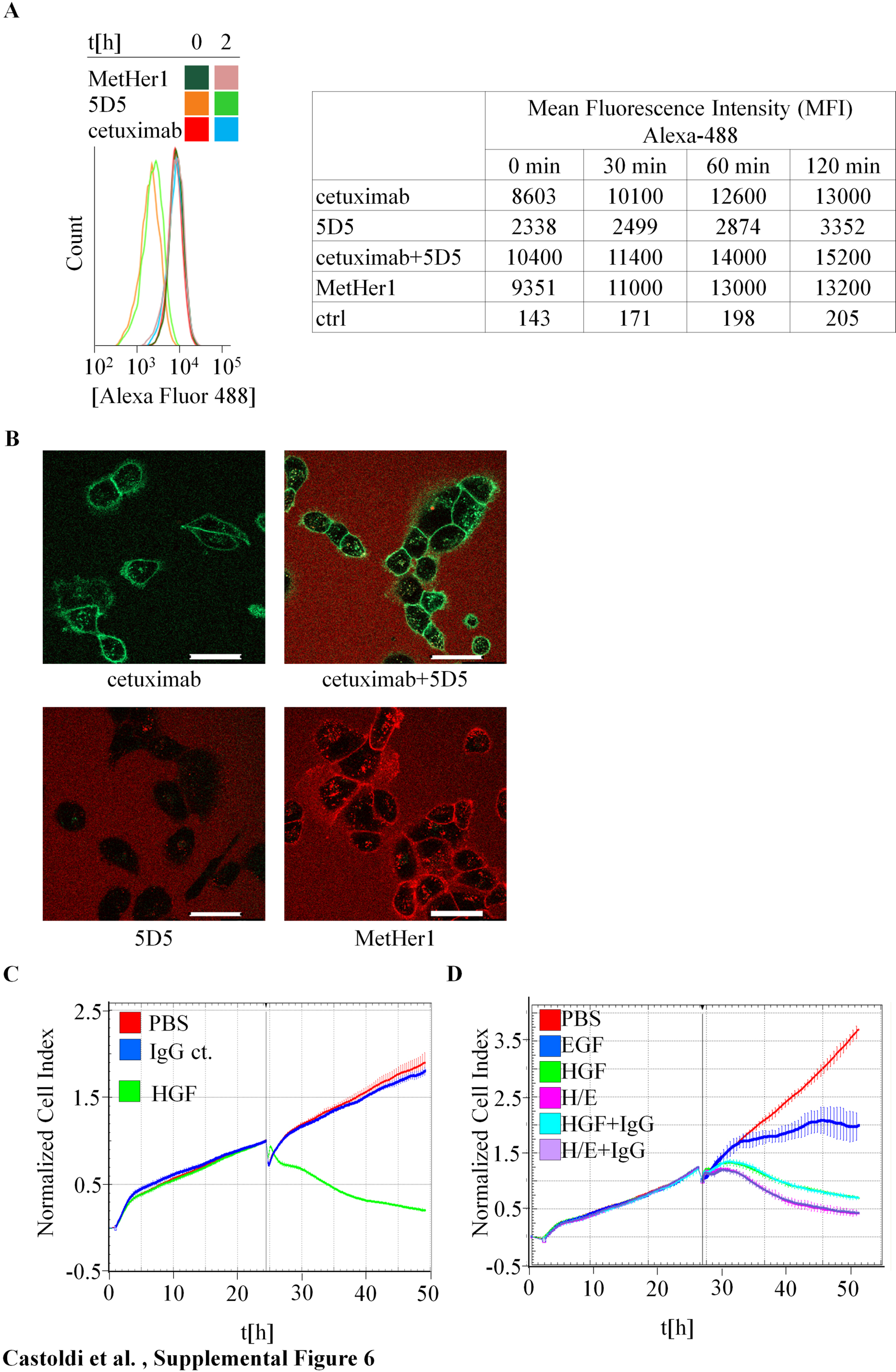

Supplement: Supplementary Figure S6 [file onc2013245x6.tif]

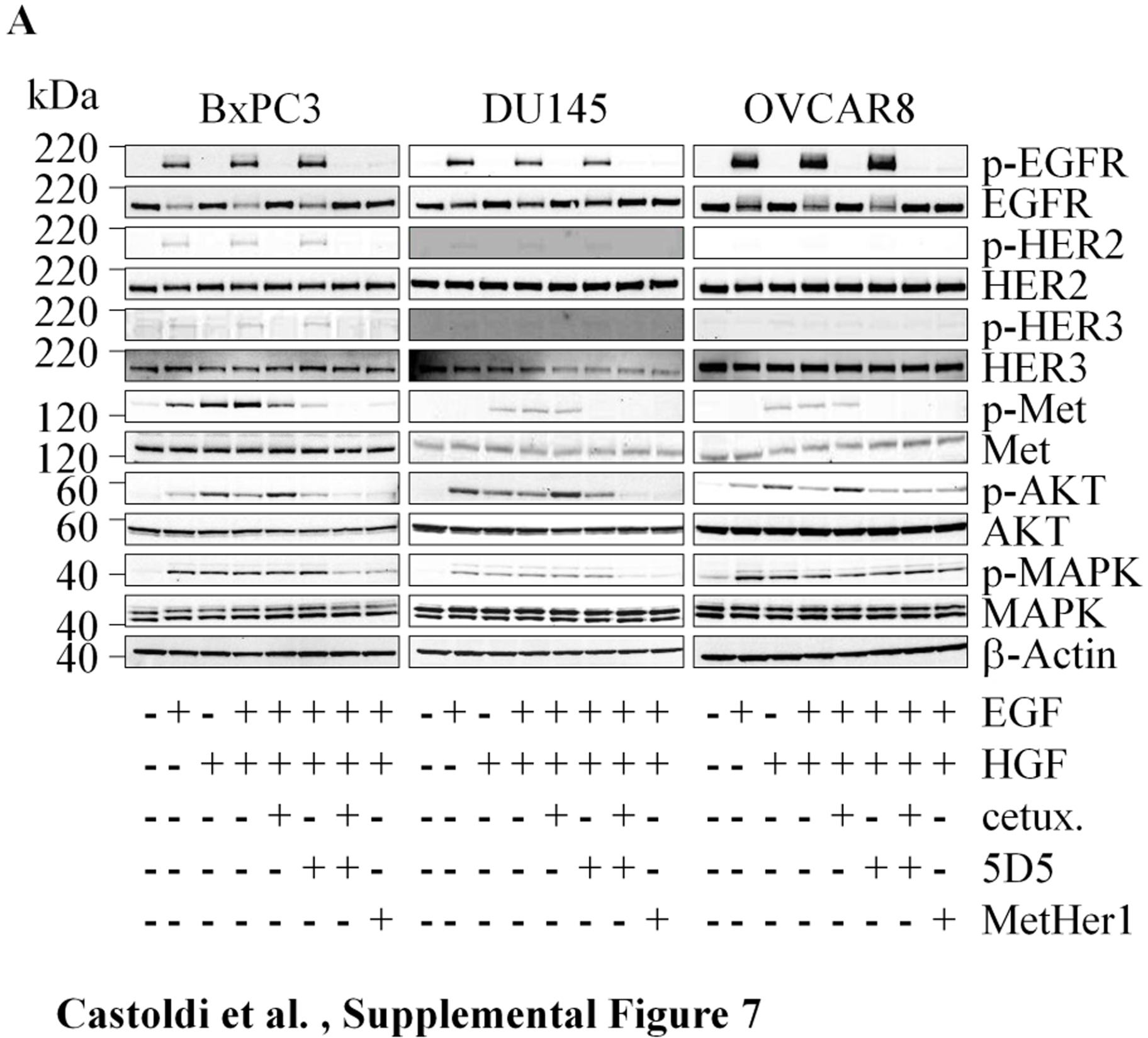

Supplement: Supplementary Figure S7 [file onc2013245x7.tif]

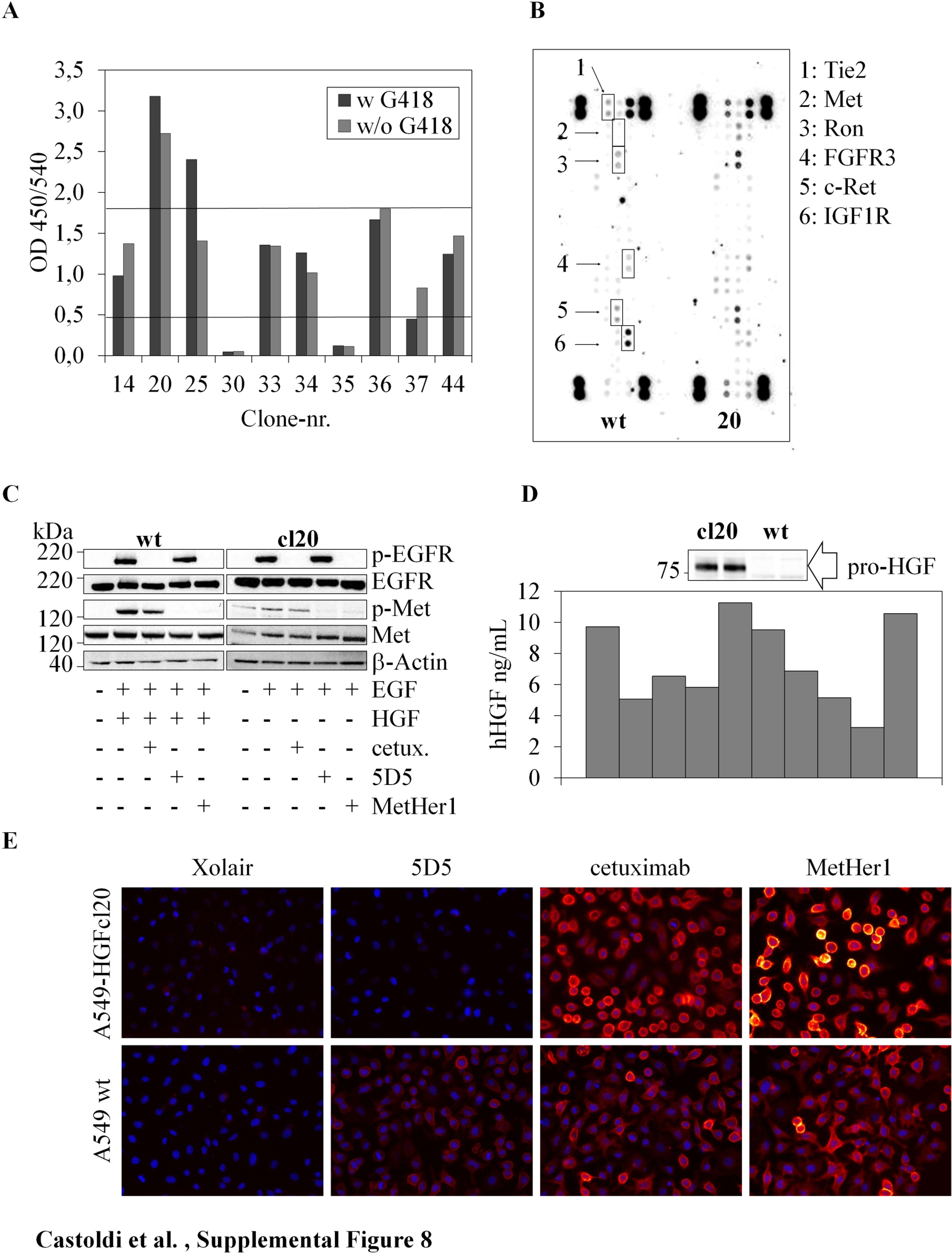

Supplement: Supplementary Figure S8 [file onc2013245x8.tif]
