# Supplementary material for: A novel bispecific EGFR/Met antibody blocks tumor-promoting phenotypic effects induced by resistance to EGFR inhibition and has potent antitumor activity
Source: Oncogene. 2013 Jul 1;32(50):5593–601. doi: 10.1038/onc.2013.245 (PMC3898114; doi:10.1038/onc.2013.245)
Supplement: Supplementary Information [file onc2013245x9.doc]

**Castoldi et al.:** Supplemental Data for “A novel bispecific EGFR/Met antibody blocks tumor promoting phenotypic effects induced by resistance to EGFR inhibition and has potent anti-tumor activity”

## Supplemental Figure Legends

## Supplemental Figure S1

(**A, B**) Analysis of AKT phosphorylation upon treatment of H596 and A431 cells with the indicated antibodies. HGF and EGF stimulation was used as positive control. (**C**) Analysis of cetuximab treatment in H596 in the presence of one or both growth factors. Numbers below the immunoblot indicate relative quantitation of signals with HGF treatment only set to 1.0.

## Supplemental Figure S2

(**A**) Cytotoxicity is presented versus untreated A431 after 43h of stimulation with HGF and EGF together with the indicated treatments. Cytotoxicity was directly determined in medium obtained from matrigel chambers before cell fixation. 3 µM Staurosporine was used as positive control for the assay. (**B**) Immunoblot of phosphorylated and total AKT in A431 cells stimulated for 10 min with HGF and EGF after 100 min treatment with two concentrations of AKT inhibitor (0.5 and 1μM).

## Supplemental Figure S3

Generation of MetHer1. (**A**) Schematic presentation of MetHer1. KiH and disulfide bridges are indicated. scFab: V_L_-C_L_-(G_4_S)_6_GG-V_H_-C_H1_, fusion via a (G_4_S)_3_ connector. (**B**) SDS-PAGE analysis of purified MetHer1 under non-reducing (NR) and reducing (R) conditions. (**C**) Size exclusion purification of MetHer1. (**D**) Analytical HPLC of MetHer1. (**E**) Simultaneous binding to recombinant Met and EGFR (Biacore). EGFR was amine-coupled to the chip surface. (**F**) Immunoblot analysis of Met phosphorylation by indicated antibodies in H596 after 30 min of incubation.

## Supplemental Figure S4

SPR analysis of binding kinetics. (**A**) Concentration series of soluble receptor binding to the respective antibodies (cetuximab, 5D5, MetHer1). Sensorgrams were fitted to a Langmuir 1:1 model, RI=0 (black lines). (**B**) Kinetic constants for soluble receptor binding to cetuximab, 5D5 and MetHer1.

## Supplemental Figure S5

(**A**) HGF concentrations in medium upon cultivation of H596 with or without lung normal fibroblasts (LNF) or lung tumor fibroblasts (LTF) for two (D2) and six (D6) days. All cells were maintained in the same volume of medium. (**B**) Percentage growth inhibition versus control of BxPC3 and H596 cell lines after treatment with a sub-optimal dose of cisplatin alone or in combination with MetHer1.

## Supplemental Figure S6

(**A**) *Left:* Internalization of indicated antibodies measured by FACS as DU145 cell surface binding after two hours of incubation at 37°C. *Right:* Mean fluorescence intensity (MFI) values of the same experiment including also the time points 30 and 60 min. (**B**) Internalization of fluorescently-labeled antibodies evaluated in DU145 cells with confocal microscopy after 4h of incubation (white bar x,y: 50µm). (**C**) RTCA-based analysis of DU145 scattering in presence and absence of HGF. Addition of human IgG control in the absence of HGF was compared to PBS. (**D**) Effect of human IgG control antibody (0.2μM) on scattering induced by HGF and HGF+EGF in DU145 cells measured by RTCA.

## Supplemental Figure S7

(**A**) Expression and phosphorylation of EGFR, HER2, HER3, Met, and the downstream signaling proteins MAPK and AKT in BxPC3, DU145, OVCAR8 cells after treatment with MetHer1, and parental antibodies cetuximab and 5D5 in the presence of HGF and EGF stimulation. This figure contains data already presented in Figure 4D and 5B.

## Supplemental Figure S8

(**A**) Relative HGF amounts in the culture medium of A549 clones (numbers x-axis) stably transfected with human HGF after 29 days of *in vitro* culture. Cells maintained HGF expression in presence (black) and absence (grey) of neomycin. (**B**) Phospho-RTK array of HGF-transfected A549 clone20, versus A549 wild type (wt) cells. Receptors with visible differences in phosphorylation are shown in the squares. (**C**) Expression and phosphorylation of Met and EGFR in HGF-transfected A549 clone20, with respect to wild type A549 after treatment with MetHer1, and parental antibodies cetuximab and 5D5. (**D**) Human HGF levels in 10 untreated animals from the HGF-overexpressing subcutaneous A549 human lung adenocarcinoma xenograft model. *Ex vivo* immunoblot analysis of tumor lysates confirmed presence of HGF in A549 clone20 but not in wild type (wt) cells. (**E**) *In vitro* binding of Cy5-labeled MetHer1, cetuximab and 5D5 to A549 clone20 versus A549 wild type cells.

## Supplemental Data: Materials and Methods

### Co-cultures

H596 were seeded as mono-culture (5000 cells/well) or in co-culture with lung normal and tumor (adenocarcinoma) primary fibroblasts - 2000 cells/well with 3000 cells/well fibroblasts - in 5% PANEXIN NTA RPMI medium in 96 well-polyhema-coated plates, where cells could form spheroids and grow in suspension. Cells were treated with 30μg/mL antibodies and viability was measured after 5d.

### Confocal microscopy

DU145 (1x10^4^/well) were seeded in ibidi 8-well μ slides and treated the following day either for 24h with HGF (30ng/mL) or for 4h with 10μg/mL cetuximab-Alexa488, 5D5-Cy5, MetHer1-Cy5. Images were acquired by confocal laser scanning microscope (Nikon Eclipse TE-2000-E, Nikon D-Eclipse C1, 4 Laser System). Calcein (3μg/mL) was added 30min before analysis at T=37°C.

### Surface Plasmon Resonance

Experiments were performed with a Biacore T200 instrument via standard amine-coupling to EDC/NHS activated chip surfaces. PBS 0.05% (v/v) Tween20 was used as running buffer and dilution buffer (with further addition of 1mg/mL BSA). For kinetic characterization of single antigen binding to MetHer1, the bispecific and parental antibodies were captured with an amine-coupled goat anti-human IgG (CM5 chip). Dilution series (c ~ 4-1200nM Met, EGFR) were analyzed in duplicates with an association phase of 180s and a dissociation phase of 1200s, at T=37°C and with a flow rate of 50μL/min. Signals were double referenced against a flow cell containing only dilution buffer. Kinetic constants were calculated from fitting to Langmuir 1:1 model (RI=0). For simultaneous binding evaluation, MetHer1 antibody (c=15nM) was captured by amine-coupled EGFR. Met (c=200/400nM) was subsequently injected at 25°C to minimize dissociation.

### Relative quantitation of HGF

Relative quantitation of HGF levels out of serum from mice or cell culture medium was performed with a HGF ELISA (R&D Systems). Experiments were carried out as recommended by the manufacturer. Different dilutions were measured against an internal calibrator standard provided by the kit.

### Proliferation Assay (MetHer1 + Cisplatin)

BxPC3, 2500 cells/well, and H596, 5000 cells/well, were seeded in medium with 10% FCS and treated the following day with 0.2μM MetHer1 for 15 min before stimulation with 30ng/mL HGF. Cisplatin (7μM and 14μM for BxPC3 and H596 respectively) was added 48h prior to measuring. Viability was evaluated via Cell Titer Glo, according to the manufacturer’s specifications (Promega) 5 days after treatment.

### FACS internalization assay

DU145 cells (5x10^5^) were diluted in 50μL of the different antibody solutions (10μg/mL) and incubated at 37°C for 30, 60 or 120 min. Cells were kept on ice and stained with 5μg/mL anti-human IgG AlexaFluor-488 (Invitrogen). Samples were fixed and measured (BD, FACS Canto).

### Generation of A549 cell line stably expressing human HGF

The coding sequence of human hepatocyte growth factor was cloned in the retroviral pLXSN vector (Clontech). The pLXSN vector contains a neomycin cassette which allows selection of infected cells. Virions were generated by transient transfection of this plasmid with Fugene HD (Roche) in the PA317 (ATCC, CRL-9078) cell line. Cell supernatants were harvested after 3d and sterile filtered through 0.45µm cellulose acetate filters (Nalgene). A549 cells were seeded at 1x10^6^ cells per 10 cm cell culture plate and allowed to attach overnight. The following day, medium was replaced by 4 mL of a logarithmic dilution series of the cell supernatant containing virions in medium containing 8µg/mL polybrene (Millipore). After 24h of infection, cells were washed and fresh complete medium without virions or polybrene was added. After additional 48h, selection medium containing 0.5 mg/mL G418 (Life Technologies) was added. Stable clones were picked using cloning rings and maintained under G418 selection. Absence of virion particles was confirmed by PCR on reverse transcribed mRNA of isolated clones.

### Phospho-RTK array with stably expressing HGF A549 clones

A549 clones were maintained in medium containing 0.5 mg/mL G418. 80-90% confluent clones were harvested by detachment of cells with Accutase (Invitrogen). Cells were lysed in buffer containing 1% NP-40, 20mM Tris-HCl (pH 8.0), 137mM NaCl, 10% glycerol, 2mM EDTA, 1mM sodium orthovanadate and protease inhibitors. 100µg of cell lysate, as quantified by the BCA method, were incubated overnight with a phospho RTK membrane (R&D Systems). Detection and image acquisition was performed as recommended by the manufacturer.

### Labeling of antibodies

Fluorescently labeled antibodies were obtained by lysine linker chemistry. The antibody to fluorophore ratio was about 1:3 for all antibodies. Before use, fluorescently labeled antibodies were evaluated in Biacore to confirm that binding properties were unaltered.

### Imaging

Tumor bearing SCID beige mice were injected i.v. with 50µg of Cy5-labeled Xolair and cetuximab, 33.3µg of 5D5 and 66.7µg of MetHer1 to assure equal molarity. NIRF signal was measured 48h after i.v. injection with the Maestro System (CRI) at optimal acquisition times. Images were processed and normalized to obtain optimal comparability.

### HGF-expressing A549 clone20 and A549 wild-type in vitro staining

Wild type and HGF-expressing A549 clone20 cells were seeded at a concentration of 2x10^6^ cells/mL into µ-slides VI (ibidi). After 24h, cells were washed with PBS and incubated for 30 min with 50 µl of 2.5 mg/mL Cy5-labeled antibodies, as reported; nuclei were subsequently stained with 50 µl of a HOECHST33342 solution (10µg/ml) for 15 min. Slides were imaged multi-spectrally with the Nuance-System (CRi) and analyzed. The displayed pictures were normalized for optimal comparability.
